# Characterization of Psychiatric Adverse Events Reported with Levetiracetam in the FDA Adverse Event Reporting System (FAERS)

**DOI:** 10.3390/healthcare14203546

**Published:** 2026-10-21

**Authors:** Suad Alshammari, Monther Alsultan, Amjad Albuobayd, Fatemah Buhaya, Hajar Alsaqer, Fatima Al-Ali, Sara Abudahab, Dayanjan Wijesinghe, Sulaiman Almohaish

**Affiliations:** 1 Department of Clinical Pharmacy, College of Pharmacy, Northern Border University, Rafah 91911, Saudi Arabia; suad.hamed@nbu.edu.sa; 2 College of Clinical Pharmacy, King Faisal University, Al-Ahsa 31982, Saudi Arabia; 3 Department of Pharmacotherapy and Outcomes Science, School of Pharmacy, Virginia Commonwealth University, Richmond, VA 23298, USA

**Keywords:** levetiracetam, antiseizure medications, psychiatric adverse events, pharmacovigilance, FAERS, epilepsy, antidepressants, antipsychotics

## Abstract

**Background:** Levetiracetam (LEV) is among the most frequently prescribed antiseizure medications, yet its tolerability is limited in some patients by psychiatric and behavioral adverse events. Because psychiatric comorbidity and psychotropic co-medication are common in epilepsy, attributing such events to LEV rather than to the underlying disease remains difficult. Thus, we sought to characterize LEV-associated psychiatric reporting and to determine whether it differs by age, sex, and concurrent psychotropic use. **Methods:** Different safety reports submitted to the FDA Adverse Event Reporting System (FAERS) from 2013 through 2022 were extracted and joined by case identifier, restricted to LEV as the primary suspect drug, with co-administered antidepressants and antipsychotics flagged. Reports lacking age or sex were excluded. Groups were compared using the *t*-test and the chi-square or Fisher exact test, with odds ratios referenced to LEV without a recorded antidepressant or antipsychotic. **Results:** Among 928 reports (mean age 48.0 ± 20.1 years; 60.9% female), dizziness (30.1%), anxiety (16.5%), agitation (10.3%), insomnia (9.8%), depression (9.6%), and amnesia (8.8%) accounted for more than 85% of adverse events. The profile was age dependent. Agitation was reported more often for adolescents than for adults (adjusted OR 3.22, 95% CI 1.67–6.20). Psychotropic co-medication was recorded in 230 reports (24.8%). Furthermore, anxiety (OR 2.07, 95% CI 1.43–2.99) and depression (OR 1.82, 95% CI 1.15–2.89) were reported more frequently in co-medicated reports, whereas dizziness (OR 0.59, 95% CI 0.42–0.84) and agitation (OR 0.44, 95% CI 0.25–0.83) were reported less frequently. The association with agitation was attenuated and was no longer statistically significant after adjustment (aOR 0.61, 95% CI 0.33–1.14). **Conclusions:** These findings characterize age- and co-medication-related differences in psychiatric adverse-event reporting with levetiracetam. After correction for multiple comparisons, agitation remained more frequently reported among adolescents, while other apparent age-related differences were not statistically significant. These findings provide descriptive pharmacovigilance evidence regarding psychiatric adverse-event reporting patterns associated with levetiracetam.

## 1. Introduction

Epilepsy affects approximately 1% of the global population, and psychiatric comorbidities occur in around 13–37% of patients [1]. Management of epilepsy therefore involves more than seizure control, as psychiatric symptoms may influence treatment response, adherence, quality of life, disease burden, and overall patient outcomes. Consequently, the adverse effect profile of antiseizure medications has become an important consideration in treatment selection.

Among antiseizure medications (ASMs), levetiracetam (LEV) is one of the most commonly prescribed and is in fact recommended as a first-line therapy in several clinical guidelines [2,3]. Its widespread use is mainly due to its efficacy across different seizure types, favorable pharmacokinetic properties including rapid onset of action, and limited drug–drug interactions increasing its safety profile. However, LEV-associated central nervous system adverse effects (CNS-AEs) include a broad spectrum of symptoms, ranging from psychiatric and behavioral disturbances, such as irritability, agitation, anxiety, depression, insomnia, psychosis, and suicidal ideation, to neurologic and cognitive effects, including dizziness, vertigo, amnesia, and disorientation [4,5]. Therefore, these effects may interfere with treatment and, in some reports, result in discontinuation of therapy [4,5].

Assessing the psychiatric safety profile of LEV is complicated by the underlying predisposition of individuals with epilepsy to psychiatric disorders, as well as potential confounders that may influence the observed associations. Depression, anxiety, and behavioral symptoms occur more frequently among patients with epilepsy compared with the general population, reflecting perhaps to the effects of seizure activity, neurological dysfunction, psychosocial factors, and other clinical characteristics that may contribute to these psychiatric outcomes [6]. As a result, determining whether psychiatric symptoms are related to LEV exposure or represent manifestations of the underlying disease remains difficult, yet important, in clinical practice.

Several studies have examined behavioral outcomes associated with LEV, although estimates vary according to patient population, study design, and methods used to assess adverse events. One study reviewed 42 observational studies and reported clinically relevant behavioral symptoms among LEV-treated patients, including irritability in 9.9% and aggression in 2.6% of patients. Irritability was associated with treatment discontinuation in 3.4% of reports [7]. In 2026, a research group identified neuropsychiatric events associated with LEV exposure, including irritability, aggression/agitation, anxiety, and behavioral changes, and reported higher rates of somnolence and fatigue compared with placebo [8]. Moreover, another study described irritability, aggression, and agitation as frequently reported behavioral manifestations, while severe events such as psychosis and suicidality occurred less commonly [9]. Similar findings have been reported in pediatric populations. In 2014 a study observed increased hostility, nervousness, and aggression among children receiving LEV compared with placebo, although few patients discontinued treatment because of these effects [10].

Various patient-related characteristics have been suggested as factors related to the occurrence of psychiatric and behavioral adverse events (PBAEs) on LEV. As mentioned earlier, a previous history of psychiatric illness is one of the best predictors of PBAEs; other factors include young age, male sex, poorly controlled epilepsy, secondary generalised epilepsy, and absence seizures [9]. Yet the results from different studies are not consistent, and it has not been determined how much individual demographic and clinical features may contribute. Moreover, since psychiatric disorders are common among people with epilepsy, many individuals are also given antidepressant/antipsychotic treatment monotherapies as well as combination therapies which makes it more difficult to interpret psychiatric side effects [11]. The existence of a pre-existing mental disorder together with the use of psychotropic drugs might affect the frequency, severity, or reporting of adverse events associated with LEV, making it hard to tell whether the effects are due to the medication or are manifestations of the original condition. It may therefore be essential to first understand demographic features and the use of other medications in real-world populations to be able to identify patients at greater risk and improve treatment selection on an individual basis as well as clinical monitoring.

Pharmacovigilance databases capture adverse event reports from routine clinical practice and enable the assessment of reporting patterns across large and diverse patient populations. Although these data cannot establish causality, they can identify potential safety signals and patient characteristics associated with increased reporting. In this study, we analyzed data from the FDA Adverse Event Reporting System (FAERS) from 2013–2022 to evaluate real-world reporting patterns of LEV-associated psychiatric and behavioral adverse events. We examined demographic factors, including age, sex, and geographic region, to characterize the patient groups in which these events were most frequently reported. Because psychiatric conditions are common among patients with epilepsy and many reports receive concurrent psychotropic medications, we also assessed whether antidepressant or antipsychotic use influenced LEV-associated reporting patterns. Finally, we evaluated the most frequently reported psychiatric and behavioral adverse events associated with LEV exposure.

This analysis adds to existing evidence on LEV-related psychiatric safety by examining adverse event reports alongside patient characteristics and concomitant medication use within a large pharmacovigilance database. The findings may help clinicians recognize patients who require closer monitoring and support decisions regarding ASM selection and follow-up.

## 2. Methods

### 2.1. Data Source and Study Design

**Figure 1:**
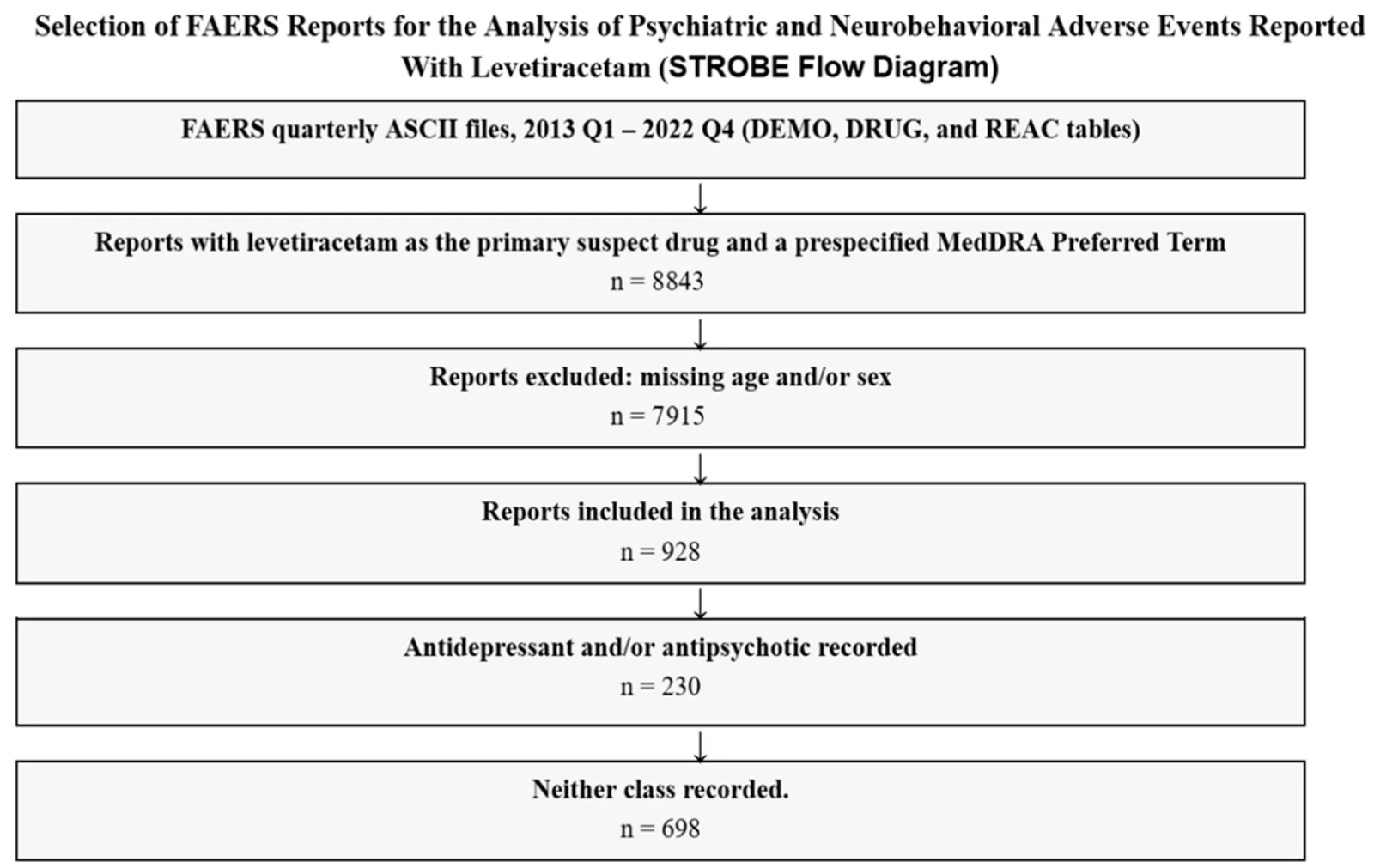
Flow diagram of the identification and inclusion of FAERS reports in which levetiracetam was the primary suspect drug. Note: counts refer to FAERS reports.

We conducted a retrospective, descriptive analysis of spontaneous reports submitted to the United States Food and Drug Administration (FDA) Adverse Event Reporting System (FAERS). FAERS is a publicly available pharmacovigilance database that collects reports of adverse events, medication errors, and product-quality complaints submitted by healthcare professionals, consumers, and manufacturers in support of the FDA post-marketing safety surveillance program. The quarterly FAERS data files covering the first quarter of 2013 through the fourth quarter of 2022 were downloaded from the FDA website (https://fis.fda.gov, accessed on 10 October 2023) in 2023. The study period was predefined to include FAERS reports available through December 2022 at the time of data extraction and initiation of the analysis. Each quarterly release is provided in ASCII and XML formats; we used the ASCII files, which comprise linked tables of demographic (DEMO), drug (DRUG), reaction (REAC), outcome (OUTC), report source (RPSR), therapy (THER), and indication (INDI) information. Reporting follows the STROBE statement where applicable, and the selection of reports is shown in Figure 1.

### 2.2. Data Extraction and Cleaning

Data extraction and integration were performed in 2023 using the KNIME Analytics Platform, version 5.3.2 (KNIME AG, Zurich, Switzerland). A published KNIME workflow for processing FAERS data was adapted to the objectives of this study [12]. The original workflow reads the quarterly FAERS files, links the demographic, drug, and reaction tables, and groups reports by medication. We modified it in three ways. First, levetiracetam reports were identified in the DRUG table using the DRUGNAME field together with the corresponding active ingredient field (PROD_AI = levetiracetam), which captured all brand and generic product names (e.g., Keppra, Keppra XR, E Keppra, and generic levetiracetam products). Drug role was taken from the ROLE_COD variable, which the FDA FAERS data dictionary defines as primary suspect (PS), secondary suspect (SS), concomitant (C), or interacting (I); reports in which levetiracetam was coded as the primary suspect drug (ROLE_COD = PS) were retained. Second, co-reported antidepressants and antipsychotics were identified in the DRUG table using predefined lists of active ingredients and brand names (Supplementary Table S1) and flagged in each report, irrespective of their reported role; the flagged drugs were reviewed manually, and non-psychotropic drugs captured by name matching were removed. Third, the analysis was restricted to reports containing a prespecified MedDRA Preferred Term (PT), described below. The quarterly outputs were then concatenated into a single dataset. Tables were linked using PRIMARYID, the unique identifier of each report in FAERS. When a FAERS report contained more than one qualifying MedDRA Preferred Term, a single term was retained for the event-specific analysis. This approach ensured that each report contributed only once to the overall event-frequency analysis.

FAERS reports may contain multiple versions of the same case as follow-up information is submitted to the FDA. The CASEID variable was used to identify reports belonging to the same case, while CASEVERSION was used to distinguish successive versions. For cases with more than one version, only the record with the highest CASEVERSION, representing the most recent follow-up report, was retained. The PRIMARYID corresponding to the retained case version was subsequently used to link information across the FAERS demographic, drug, reaction, indication, therapy, and outcome files. Earlier versions of the same case were excluded to prevent duplicate counting.

### 2.3. Adverse Event Terms and Classification

Seventeen MedDRA Preferred Terms were prespecified: agitation, amnesia, anxiety, delusion, depression, discomfort, disorientation, dizziness, hostility, impatience, insomnia, mood swings, nervousness, panic disorder, restlessness, social anxiety disorder, and vertigo. Sixteen of these were recorded among the included reports. PTs were used as coded by the FDA in the FAERS REAC files. Because the FDA codes each quarterly release with the MedDRA version current at that time, the terms span the MedDRA versions in use from 2013 to 2022 and no single version applies. Because not all of these terms are psychiatric, each PT was classified by its MedDRA primary System Organ Class (SOC) and grouped into clinical categories: anxiety and mood (anxiety, depression, mood swings, nervousness, panic disorder, social anxiety disorder), behavioral (agitation, restlessness, hostility, impatience), sleep (insomnia), psychotic (delusion), cognitive (amnesia, disorientation), neurological or vestibular (dizziness, vertigo), and nonspecific (discomfort) (Supplementary Table S1). The extraction workflow retained one qualifying PT per report; each report therefore contributes a single PT, and the PT frequencies are mutually exclusive.

### 2.4. Variables

Variables of interest were defined prior to data analysis. The ‘age group’ variable was used and defined as children (1–12 years of age), adolescents (13–17 years of age), adults (18–64 years of age) and the elderly (65+ years of age). The variable of ‘patient’s body weight’ was also recorded in the relevant reports. Other variables of interest were the ‘primary drug of interest’ which was recorded as levetiracetam (and referred to by the brand name Keppra where appropriate) and the ‘concomitant use of other antidepressants and/or antipsychotics’. Other variables of interest included the ‘psychiatric adverse-event term(s) reported’ in relevant reports. Prochlorperazine, a typical phenothiazine that is frequently used as an antiemetic rather than for a psychiatric indication, was classified as a typical antipsychotic and examined in a sensitivity analysis.

### 2.5. Inclusion and Exclusion Criteria

Reports were included if levetiracetam was reported as the primary suspect drug together with a prespecified PT. Reports without a recorded age or sex were excluded. The extraction identified 8843 levetiracetam reports with a prespecified PT, of which 7915 lacked age and/or sex; the remaining 928 reports were analyzed (Figure 1). In the extracted dataset, missing age, sex, and body weight co-occurred almost entirely, so all 928 included reports had a recorded body weight.

### 2.6. Statistical Analysis

Analyses were conducted in Python (version 3.11, Python Software Foundation, Beaverton, OR, USA) using pandas (3.0, NumFOCUS, Inc., Austin, TX, USA), SciPy (1.17, NumFOCUS, Inc., Austin, TX, USA), and statsmodels (0.15, NumFOCUS, Inc., Austin, TX, USA). Because FAERS contains spontaneous reports without information on the number of exposed patients, all analyses describe reporting patterns among LEV reports and were not used to estimate incidence, prevalence, or risk. Reports with a recorded antidepressant, antipsychotic, or both were compared with reports without either class. Continuous variables (age and body weight) are presented as mean ± standard deviation and were compared using the independent-samples *t*-test. Categorical variables are presented as counts and percentages and were compared using the Pearson chi-square test or the Fisher exact test; the *p*-values for the individual PTsare from the Fisher exact test. For each PT, odds ratios (ORs) with 95% confidence intervals (CIs) were calculated with the group without either class as the reference; an OR above 1 indicates that the term was reported more often when psychotropic co-medication was recorded. These ORs compare two subsets of LEV reports and are not disproportionality measures (such as reporting odds ratios computed against other drugs or the full database). The distribution of PTs was also examined by sex and age group. Significance was declared at a two-sided *p* < 0.05. Addition statistical analysis were performed on the same 928 reports. First, for PTs with at least 20 reports and at least 5 exposed reports, logistic regression was used to estimate ORs for psychotropic co-medication adjusted for age (continuous) and sex. Second, differences between age groups were examined with a Monte Carlo chi-square test (10,000 permutations) and with logistic regression models for each PT that included age group (reference: adults), sex, and psychotropic co-medication. Third, to account for multiple comparisons, the Benjamini–Hochberg procedure was applied within each family of tests (16 PT comparisons by co-medication, 8 adjusted co-medication models, 24 age-group contrasts, and 16 PT comparisons by sex), and false discovery rate–adjusted q-values are reported alongside *p*-values. Fourth, three sensitivity analyses were performed: (i) reclassifying the six reports whose only recorded psychotropic was prochlorperazine as unexposed; (ii) additionally adjusting for body weight; and (iii) adjusting for age group instead of continuous age. The results are presented in Supplementary Tables S2–S4. Supplementary analyses used logistic regression adjusted for age and sex and the Benjamini–Hochberg correction for multiple testing.

### 2.7. Data Availability

FAERS data are publicly available from the FDA. The Python analysis code, the drug lists, the PT list, and the list of FAERS identifiers of the included reports are provided as Supplementary Materials.

## 3. Results

This analysis included 928 FAERS reports in which levetiracetam was the primary suspect drug and a prespecified PT was recorded, submitted between 2013 and 2022 (Figure 1). Of the 8843 reports identified by the extraction, 7915 were excluded because age and/or sex was missing. Throughout this section, frequencies refer to reports, not to confirmed unique patients or causally confirmed events.

### 3.1. Demographic Characteristics

Table 1 summarizes the demographic characteristics of the included reports. The mean age was 48.0 years (SD 20.1), and the mean body weight, which was recorded for all 928 reports, was 74.2 kg (SD 26.0). Adults accounted for most reports (628, 67.7%), followed by older adults (209, 22.5%), adolescents (56, 6.0%), and children (35, 3.8%). Overall, females accounted for 60.9% (565) of reports and males for 39.1% (363). Female reports outnumbered male reports in every age group except children (14 vs. 21).

With respect to geographic origin, most reports were submitted from North America (533; 57.4%), followed by Europe (318; 34.3%), with the remaining reports originating from other regions (77; 8.3%); the distribution is presented in Table 2.

### 3.2. Profile of Reported Adverse Event Terms

Sixteen PTs were recorded (Table 3). Dizziness was the most frequently reported term (279; 30.1%), followed by anxiety (153; 16.5%), agitation (96; 10.3%), insomnia (91; 9.8%), depression (89; 9.6%), and amnesia (82; 8.8%). Together, these six terms accounted for more than 85% of reports. The remaining terms (disorientation, vertigo, discomfort, mood swings, restlessness, nervousness, delusion, hostility, impatience, and panic disorder) were reported less frequently. When grouped by category, neurological or vestibular terms (dizziness and vertigo) accounted for 33.8% of reports, anxiety and mood terms for 28.8%, cognitive terms for 12.8%, behavioral terms for 12.1%, and insomnia for 9.8%; terms within the MedDRA psychiatric disorder SOC accounted for 513 reports (55.3%) (Supplementary Table S1).

### 3.3. Reported Adverse Events by Age Group

The distribution of reported terms within each age group is shown in Table 4, and the complete distribution of all 16 terms is given in Supplementary Table S2. Among adults, dizziness was the most frequently reported term (183; 29.1%), followed by anxiety (116; 18.5%), insomnia and depression (60 each; 9.6%), and agitation (59; 9.4%). Older adults showed a similar distribution, with dizziness (79; 37.8%) more frequent than anxiety (30; 14.4%) and amnesia (21; 10.0%) (Table 4).

The profile differed in the younger age groups. Among adolescents, agitation was the most frequently reported term (16; 28.6%), followed by dizziness (14; 25.0%) and depression (9; 16.1%). Among children, agitation (9; 25.7%) and insomnia (8; 22.9%) were most frequent.

In the supplementary analysis, the distribution of terms differed across age groups (Monte Carlo chi-square *p* < 0.001). After adjustment for sex and psychotropic co-medication, agitation was reported more frequently for adolescents than for adults (adjusted OR [aOR] 3.22, 95% CI 1.67–6.20; q = 0.011). Agitation (aOR 2.56, 95% CI 1.13–5.83; *p* = 0.025) and insomnia (aOR 2.87, 95% CI 1.22–6.74; *p* = 0.015) in children and dizziness in older adults (aOR 1.48, 95% CI 1.06–2.07; *p* = 0.020) were also reported more frequently than in adults, and dizziness was reported less frequently in children (aOR 0.21, 95% CI 0.06–0.71; *p* = 0.012), but these differences did not remain significant after correction for 24 age-group comparisons (q = 0.119 for each). Amnesia was reported at a similar frequency in older adults and adults (aOR 1.11, 95% CI 0.65–1.88) (Supplementary Table S2). The comparisons for children (*n* = 35) and adolescents (*n* = 56) rest on small numbers and should be interpreted with caution.

### 3.4. Reported Adverse Event by Sex

Agitation was reported more frequently for males than for females (54/363, 14.9% vs. 42/565, 7.4%; *p* < 0.001, q = 0.004). Dizziness (33.1% vs. 25.3%; *p* = 0.012) and vertigo (5.1% vs. 1.7%; *p* = 0.007) were reported more frequently for females, but these differences did not remain significant after correction for multiple testing (q = 0.064 and 0.053; Supplementary Table S3).

### 3.5. Adverse Events with Concurrent Antidepressant and Antipsychotic Therapy

**Figure 2:**
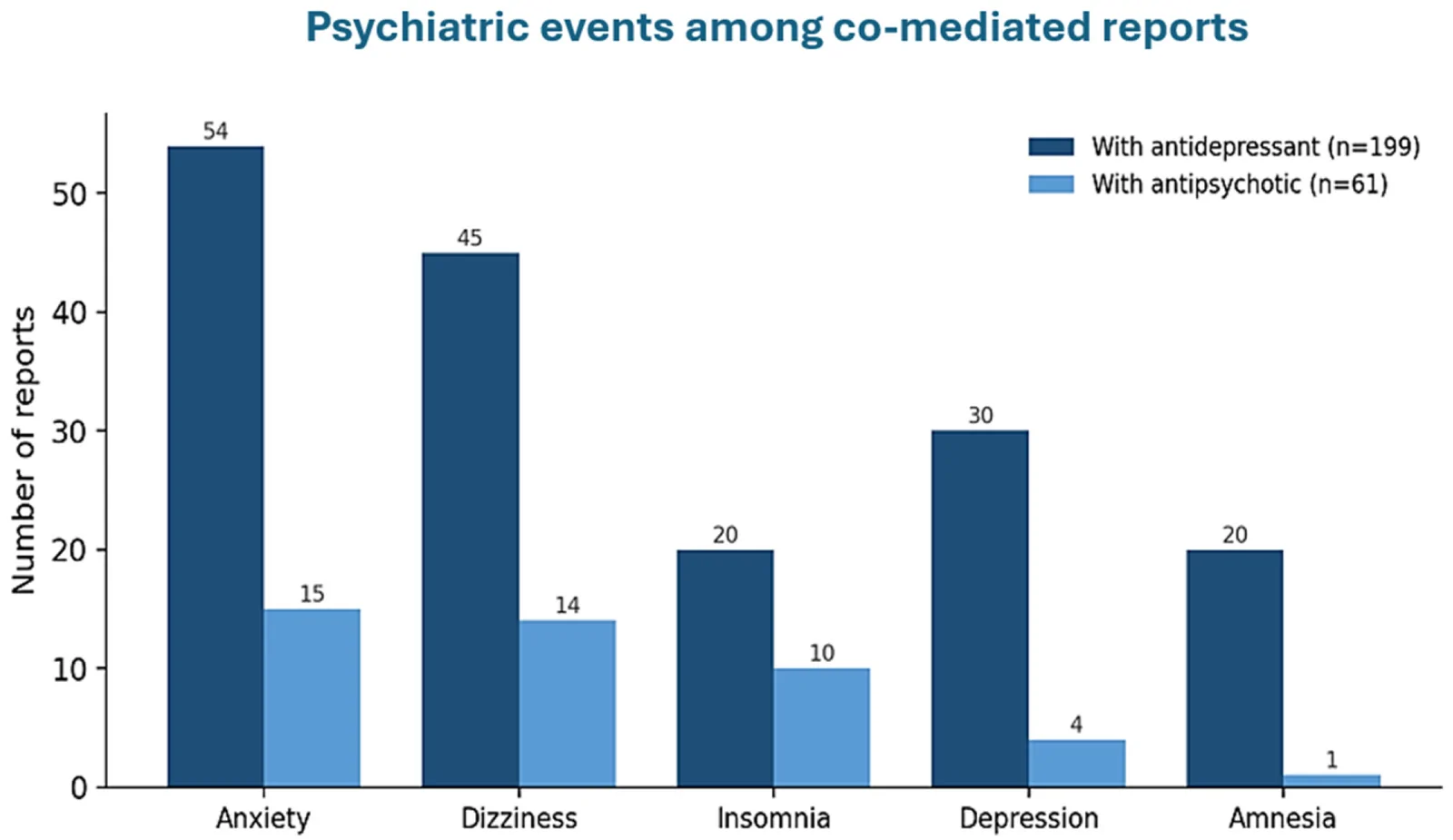
Leading psychiatric adverse events among reports co-medicated with an antidepressant or an antipsychotic, showing anxiety as the predominant event in both subgroups.

Overall, an antidepressant or antipsychotic was recorded in 230 reports (24.8%): an antidepressant only in 169, an antipsychotic only in 31, and both in 30 (Table 5). The profile of reported terms differed from that of the full dataset (Figure 2). Whereas dizziness was the most frequently reported term overall, anxiety was the most frequent term among reports with an antidepressant or an antipsychotic. Among the 199 reports with an antidepressant, anxiety was reported more often than dizziness (54 vs. 45 reports), and a similar pattern was seen among the 61 reports with an antipsychotic (15 vs. 14 reports). Among the 31 reports with an antipsychotic only, disorientation (8 reports) and insomnia (7 reports) were most frequent. Reports receiving concomitant psychotropic medications had a higher proportion of reports involving anxiety and depression compared with those not receiving psychotropic medications. These findings represent descriptive associations within the FAERS dataset and should not be interpreted as evidence of a causal or modifying effect of psychotropic treatment.

Among the 199 reports with a recorded antidepressant, the classes were selective serotonin reuptake inhibitors (100 reports; 50.3%), serotonin–norepinephrine reuptake inhibitors (46; 23.1%), tricyclic antidepressants (22; 11.1%), serotonin modulators (16; 8.0%), and atypical antidepressants (15; 7.5%). No monoamine oxidase inhibitors were recorded (Table 6). Among the 61 reports with a recorded antipsychotic, atypical agents (45 reports; 73.8%) were more frequent than typical agents (16 reports; 26.2%) (Table 7). The most frequently recorded agents were quetiapine (21 reports) and aripiprazole (10 reports). Prochlorperazine accounted for 10 of the 16 reports classified as typical antipsychotic use; however, as a phenothiazine commonly prescribed as an antiemetic, its presence should not necessarily be interpreted as reflecting treatment for a psychiatric indication. Because several medication classes contained relatively few reports, the most frequently reported adverse-event term within each class is presented descriptively and was not subjected to statistical testing.

A comparison of 698 reports treated with levetiracetam without a recorded antidepressant or antipsychotic and 230 reports treated with levetiracetam in combination with psychotropic medications is presented in Table 8.

Reports with and without a recorded antidepressant or antipsychotic are compared in Table 8. Reports with psychotropic co-medication (*n* = 230) had a higher mean age than those without (*n* = 698) (51.8 ± 15.5 vs. 46.7 ± 21.3 years; *p* < 0.001) and a higher body weight (80.6 ± 24.9 vs. 72.1 ± 26.0 kg; *p* < 0.001), and females were more frequently represented (69.1% vs. 58.2%; *p* = 0.004).

Dizziness was reported less frequently in reports with psychotropic co-medication than in those without (22.2% vs. 32.7%; OR 0.59, 95% CI 0.42–0.84; *p* = 0.003). In the unadjusted analysis, anxiety (24.8% vs. 13.8%; OR 2.07, 95% CI 1.43–2.99; *p* < 0.001) and depression (13.9% vs. 8.2%; OR 1.82, 95% CI 1.15–2.89; *p* = 0.014) were reported more frequently in reports with psychotropic co-medication, whereas agitation was reported less frequently (5.7% vs. 11.9%; OR 0.44, 95% CI 0.25–0.83; *p* = 0.006). No significant differences were observed for insomnia (11.7% vs. 9.2%; OR 1.32, 95% CI 0.83–2.13; *p* = 0.252) or amnesia (8.7% vs. 8.9%; OR 0.98, 95% CI 0.59–1.67; *p* = 1.000). After adjustment for age and sex and correction for multiple testing, the associations remained significant for anxiety (aOR 2.12, 95% CI 1.45–3.08; q = 0.001), depression (aOR 1.78, 95% CI 1.12–2.85; q = 0.042), and dizziness (aOR 0.52, 95% CI 0.37–0.75; q = 0.001). In contrast, the association with agitation was attenuated and no longer statistically significant after adjustment (aOR 0.61, 95% CI 0.33–1.14; q = 0.24). These estimates represent within-levetiracetam reporting associations and should not be interpreted as evidence that psychotropic co-medication caused or modified these events, consistent with the concentration of agitation reports among children and adolescents, in whom psychotropic co-medication was rarely recorded (3 of 91 reports) (Supplementary Table S4).

These results were consistent in the sensitivity analyses (Supplementary Table S4). After retaining only the most recent version of each case (*n* = 928), the aORs were 1.99 for anxiety, 1.78 for depression, and 0.56 for dizziness. Reclassifying the six reports whose only recorded psychotropic was prochlorperazine as unexposed gave aORs of 2.14, 1.85, and 0.55, respectively, and adjusting for age group instead of continuous age gave 1.95, 1.87, and 0.53. With additional adjustment for body weight, the estimates were 2.01, 1.75, and 0.50; the depression estimate was significant before correction (*p* = 0.020) but not after (q = 0.054).

## 4. Discussion

This analysis includes 928 reports submitted to the FAERS database from 2013 to 2022 describing neurobehavioral adverse events associated with the use of LEV. The analysis revealed three main findings. Firstly, six terms (dizziness, anxiety, agitation, insomnia, depression, and amnesia) accounted for more than 85% of reports, and dizziness, a neurological rather than psychiatric term, was the single most frequently reported. Secondly, the profile was age dependent, with agitation and insomnia leading in children and adolescents and dizziness and amnesia predominating in adults and older adults. Finally, reports of concomitant use of antidepressants or antipsychotics (N = 230; 24.8%) were compared with reports of levetiracetam without a recorded antidepressant or antipsychotic: Compared with reports of levetiracetam without a recorded antidepressant or antipsychotic, differences in the reporting of anxiety, depression, dizziness, and agitation were observed; however, these findings were interpreted according to the adjusted analyses. In particular, the apparent lower reporting of agitation observed in the unadjusted analysis was attenuated and was no longer statistically significant after adjustment (aOR 0.61, 95% CI 0.33–1.14; q = 0.24).

Previous studies have reported irritability in approximately 10% of levetiracetam-treated patients and aggression in approximately 3% [7,8]. In the present FAERS analysis, hostility was identified in only two reports (0.2%). This difference may reflect variations in event terminology, coding, and spontaneous reporting practices. Accordingly, the relative frequencies observed in FAERS should be interpreted cautiously and should not be considered estimates of the incidence of adverse events [12].

The adverse reports with development of irritability over time and attributed their new behavior to their epilepsy or to external circumstances. Thus, reports of this symptom in a more subsumed manner, i.e., as agitation or mood swings. These findings are in line with the aforementioned findings regarding the frequency rankings from the FDA Adverse Event Reporting System (FAERS) which should be treated with caution and not be used as an approximation of the actual incidence of adverse events [13,14].

The higher reporting of agitation in adolescents is consistent with systematic reviews describing hostility, nervousness, and aggression in children and adolescents receiving LEV. The pediatric findings, however, are based on 35 children and 56 adolescents, and only the association for agitation in adolescents remained significant after correction for multiple comparisons. The trends toward more frequent reporting of agitation and insomnia in children and of dizziness in older adults should therefore be regarded as hypothesis-generating [15,16]. The observed reporting patterns among older adults may potentially be influenced by factors such as age-related changes in renal function, polypharmacy, and altered central nervous system sensitivity; however, these factors were not directly evaluated in the present study and should therefore be considered possible explanations rather than established mechanisms. None of these factors was evaluated in this study. Dizziness in older adults is nonetheless clinically relevant because of its association with falls.

Females accounted for 60.9% of reports. This proportion is similar to the female share (60.1%) of more than 15 million spontaneous reports across all drug classes worldwide, so the female predominance is more likely to reflect general differences in reporting than a sex-specific effect of LEV [17]. Within the dataset, agitation was reported more frequently for males, whereas no other sex difference remained significant after correction for multiple testing.

The more frequent reporting of anxiety and depression among reports with recorded psychotropic co-medication should be interpreted cautiously. Reports prescribed antidepressants or antipsychotics are likely to have underlying psychiatric disorders, which increase the likelihood that psychiatric symptoms are present, recognized, and reported. These differences therefore probably reflect confounding by indication rather than an effect of psychotropic treatment itself, and a pharmacodynamic interaction cannot be assessed with these data. FAERS records do not reliably capture psychiatric history or the indication for the co-medication, so this confounding could not be addressed by adjustment. Reports with psychotropic co-medication also had a higher mean age and body weight and more often concerned females, and adjustment for these characteristics did not materially change the estimates. These odds ratios should be interpreted as measures of association between characteristics of levetiracetam reports and the reporting of specific psychiatric adverse events. They do not represent comparative risk estimates, incidence measures, or evidence of a causal relationship.

The reports of anxiety and/or depression in the co-medicated reports could potentially be attributed to a pharmacodynamic interaction, but are more likely to be a result of confounding by indication. This means that the reports on psychotropic medication in the first place are likely to have an antecedent psychiatric disorder, and therefore reports of psychiatric and behavioral adverse events in reports taking LEV would be expected to increase with the history of psychiatric disorder in co-medicated reports [18,19,20]. On average, co-medicated reports were older, heavier, and the majority were female. The higher reporting of anxiety and depression among reports receiving psychotropic medications should be interpreted cautiously. Reports prescribed these medications may already have underlying psychiatric disorders, which may increase the likelihood that psychiatric symptoms are present or reported. Therefore, these observed differences may reflect confounding by indication rather than an effect of psychotropic treatment itself. Accordingly, the findings should be considered descriptive associations rather than evidence that concomitant psychotropic therapy caused or modified psychiatric adverse events associated with levetiracetam.

Although the analysis focused on reports in which levetiracetam was designated as the primary suspect drug, some reports may have included other antiepileptic drugs, benzodiazepines, or other CNS-active medications that could influence psychiatric adverse-event reporting. The present study was not designed to determine the independent contribution of these concomitant therapies, and the observed findings should therefore be interpreted as reporting associations rather than evidence of causality.

### 4.1. Strengths and Limitations

A strength of this study is its use of FAERS, which captures adverse events that occur in routine clinical practice across a wide range of populations, including older adults, children, and reports receiving psychotropic drugs, who are often under-represented in clinical trials. The selection of reports is documented, and the analytical code and report identifiers are provided to allow the analysis to be reproduced.

Analysis of spontaneous reporting systems has several limitations. First of all, adverse drug events are reported on a voluntary basis. Therefore, a spontaneous reporting system does not have a true denominator of exposed reports, which hampers estimation of the incidence of adverse drug events and assessment of causality. More specifically, for this analysis, information regarding dose, rate of titration, duration of treatment, time to onset of adverse drug events, as well as information regarding type of seizures, severity of underlying epilepsy and clinical indication for treatment of most reports in this cohort are not reported. Second, most extracted reports lacked age and sex and were excluded, which reduced the sample size and may limit generalizability if reporting completeness differed by reporter type, region, or event. The present analysis focused on the overall characterization of psychiatric adverse-event reporting rather than treatment-duration-specific patterns. Because therapy start and end dates are not consistently reported in FAERS, treatment duration could not be evaluated uniformly across reports. Numerous comparisons were made; findings that were not significant after correction for multiple testing should be regarded as exploratory. The pediatric subgroups were small. An additional limitation is that only one qualifying MedDRA Preferred Term was retained for each FAERS report. Therefore, additional psychiatric or neuropsychiatric events co-reported within the same report were not captured in the event-frequency analysis, and the frequency of some individual events may consequently have been underestimated.

Future studies incorporating more complete longitudinal exposure data could further investigate the temporal pattern of these events.

### 4.2. Future Directions

Some limitations of the current study can be addressed in future studies. First, there is a need for prospective, controlled studies to gather necessary clinical information not contained in spontaneous reporting systems (such as information on seizure type and severity of epilepsy and on current medications including dose and duration of treatment). Second, a longitudinal cohort study would be very useful to separate the effects of LEV from those of the underlying epilepsy and pre-existing psychiatric disorders. Third, further research is warranted to identify the mechanisms that contribute to the observed age and sex-dependent patterns of reported with LEV psychiatric adverse events. Fourth, integrating pharmacogenomics with neuroimaging to predict and prevent psychiatric adverse events of antiseizure medications in clinical practice is an area of future research that holds much promise. Such implementation will be an important step in achieving personalized and precise treatment of patients with epilepsy through selection of optimal antiseizure medications.

## 5. Conclusions

This study demonstrates that psychiatric and neuropsychiatric adverse events reported with levetiracetam may differ across age groups and may be influenced by concomitant psychotropic therapy. After correction for multiple comparisons, agitation remained more frequently reported among adolescents, whereas other apparent age-related differences were not statistically significant. Psychiatric adverse-event reporting also differed according to concomitant psychotropic medication use; however, these findings represent descriptive associations and should not be interpreted as evidence of causality or treatment-related risk. Further prospective studies are needed to confirm these age-related patterns, clarify the influence of concurrent psychotropic therapy, and identify patients most susceptible to LEV-associated psychiatric adverse events.

## Figures and Tables

**Table 1:** Distribution of levetiracetam-associated psychiatric and neuropsychiatric adverse event reports by age group and sex (N = 928).

Age Group	Female	Male	Total
Children	14	21	35
Adolescents	29	27	56
Adults	403	225	628
Elderly	119	90	209
Total	565	363	928

**Table 2:** Geographic distribution of reports by world region.

World Region	*n*	%
North America	533	57.4
Europe	318	34.3
Other	77	8.3
Total	928	100.0

**Table 3:** Frequency of reported psychiatric and neuropsychiatric adverse events associated with levetiracetam (percentages of the 928 reports).

Prefferred Term	*n*	% of Reports
Dizziness	279	30.1
Anxiety	153	16.5
Agitation	96	10.3
Insomnia	91	9.8
Depression	89	9.6
Amnesia	82	8.8
Disorientation	37	4.0
Vertigo	35	3.8
Discomfort	19	2.0
Mood swings	16	1.7
Restlessness	13	1.4
Nervousness	8	0.9
Delusion	6	0.6
Hostility	2	0.2
Impatience	1	0.1
Panic disorder	1	0.1

Note: Frequency of Reported Preferred Terms (N = 928). Each report contributed one preferred term, so the frequencies are mutually exclusive and sum to 928.

**Table 4:** Leading reported Preferred Terms within each age group, *n* (% of the age group).

Adverse Event	Adults	Elderly	Adolescents	Children
Dizziness	183 (29.1%)	79 (37.8%)	14 (25.0%)	3 (8.6%)
Anxiety	116 (18.5%)	30 (14.4%)	6 (10.7%)	1 (2.9%)
Agitation	59 (9.4%)	12 (5.7%)	16 (28.6%)	9 (25.7%)
Insomnia	60 (9.6%)	20 (9.6%)	3 (5.4%)	8 (22.9%)
Depression	60 (9.6%)	18 (8.6%)	9 (16.1%)	—
Amnesia	57 (9.1%)	21 (10.0%)	—	—
Mood swings	—	—	—	5 (14.3%)
Group total	628	209	56	35

The complete distribution is given in Supplementary Table S2.

**Table 5:** Recorded antidepressant and antipsychotic co-medication among the 928 reports, with the most frequently reported Preferred Term in each group.

Concomitant Therapy with Levetiracetam	Reports (*n*)	% of Cohort	Reports (*n*)|% of Reports
Antidepressant only	169	18.2	Anxiety, 42 (24.9%)
Antipsychotic only	31	3.3	Disorientation, 8 (25.8%)
Both antidepressant and antipsychotic	30	3.2	Anxiety, 12 (40.0%)
Subtotal: any concomitant psychotropic	230	24.8	Anxiety, 57 (24.8%)
Levetiracetam without either class	698	75.2	Dizziness, 228 (32.7%)
Total cohort	928	100	Dizziness, 279 (30.1%)

Note: The first three rows are mutually exclusive and sum to the subtotal (169 + 31 + 30 = 230); the subtotal plus the reports without either class equal the full study sample (230 + 698 = 928). The antidepressant and antipsychotic totals (199 and 61) include the 30 dual-therapy reports and therefore exceed the corresponding “only” rows.

**Table 6:** Antidepressant classes among the 199 reports with a recorded antidepressant, with the most frequently reported Preferred Term in each class.

Antidepressant Class	*n*	%	Most Frequent Preferred Term, *n* (% of Class)
SSRIs	100	50.3	Anxiety, 29 (29.0%)
SNRIs	46	23.1	Anxiety, 10 (21.7%)
TCAs	22	11.1	Dizziness, 6 (27.3%)
Serotonin modulators	16	8	Anxiety, 8 (50.0%)
Atypical	15	7.5	Insomnia, 3 (20.0%)
MAOIs	0	0	—
Total	199	100	

**Table 7:** Antipsychotic classes among the 61 reports with a recorded antipsychotic, with the most frequently reported Preferred Term in each class.

Antipsychotic Class	*n*	%	Most Frequent Preferred Term, *n* (% of Class)
Atypical	45	73.8	Anxiety, 13 (28.9%)
Typical	16	26.2	Disorientation, 6 (37.5%)
Total	61	100	

Typical antipsychotics comprised prochlorperazine (*n* = 10), levomepromazine (*n* = 4), and haloperidol (*n* = 2).

**Table 8:** Characteristics and reported Preferred Terms of reports without (*n* = 698) and with (*n* = 230) a recorded antidepressant or antipsychotic. Odds ratios (95% confidence intervals), reported in the text, use the reports without either class as the reference. Estimates adjusted for age and sex are given in (Supplementary Table S4).

Characteristic	LEV Without a Recorded Antidepressant or Antipsychotic (*n* = 698)	LEV + Psychotropic (*n* = 230)	*p*-Value
Age, years, mean ± SD	46.7 ± 21.3	51.8 ± 15.5	<0.001
Weight, kg, mean ± SD	72.1 ± 26.0	80.6 ± 24.9	<0.001
Female sex, *n* (%)	406 (58.2)	159 (69.1)	0.004
Preferred Term, *n* (%)			
Dizziness	228 (32.7)	51 (22.2)	0.003
Anxiety	96 (13.8)	57 (24.8)	<0.001
Agitation	83 (11.9)	13 (5.7)	0.006
Insomnia	64 (9.2)	27 (11.7)	0.252
Depression	57 (8.2)	32 (13.9)	0.014
Amnesia	62 (8.9)	20 (8.7)	1

## Data Availability

The data presented in this study are openly available in FDA Adverse Event Reporting System (FAERS) Database at https://fis.fda.gov/sense/app/95239e26-e0be-42d9-a960-9a5f7f1c25ee/sheet/7a47a261-d58b-4203-a8aa-6d3021737452/state/analysis (accessed on 31 August 2026).

## Supplementary Materials

The following supporting information can be downloaded at: https://www.mdpi.com/article/10.3390/healthcare14203546/s1, Supplementary File S1, Table S1a: Prespecified MedDRA Preferred Terms, with frequency among the 928 reports, clinical category, and MedDRA primary System Organ Class; Supplementary File S1, Table S1b: Identification and classification of levetiracetam and of recorded antidepressants and antipsychotics; Supplementary File S1, Table S2a: Complete distribution of reported Preferred Terms within each age group, *n* (% of the age group); Supplementary File S1, Table S2b: Adjusted odds ratios for each Preferred Term by age group (reference: adults), adjusted for sex and psychotropic co-medication; Supplementary File S1, Table S3: Reported Preferred Terms by sex, *n* (% of sex group); Table S4a: Reports with (*n* = 230) and without (*n* = 698) a recorded antidepressant or antipsychotic: crude odds ratios and odds ratios adjusted for age and sex; Supplementary File S1, Table S4b: Sensitivity analysis: the six reports whose only recorded psychotropic wasprochlorperazine reclassified as unexposed (224 vs. 704); Supplementary File S1, Table S4c: Sensitivity analysis: adjusted for age, sex, and body weight; Supplementary File S1, Table S4d: Sensitivity analysis: adjusted for age group and sex; Supplementary File S2: FAERS PRIMARYID and CASEID; Supplementary File S3: Python analysis code.
