# Supplementary material for: Characterization of Psychiatric Adverse Events Reported with Levetiracetam in the FDA Adverse Event Reporting System (FAERS)
Source: Healthcare (Basel). 2026 Oct 21;14(20):3546. doi: 10.3390/healthcare14203546 (PMC13703985; doi:10.3390/healthcare14203546)
Supplement: Supplementary file 1 [file healthcare-14-03546-s001.zip › Supplementary_File S1.pdf]

Supplementary Material

Characterization of Psychiatric Adverse Events Reported with Levetiracetam in the FDA Adverse Event Reporting System (FAERS)

All supplementary analyses were performed on the 928 reports of the primary analysis. Supplementary File S2 lists the FAERS PRIMARYID and CASEID of the 928 included reports. Supplementary File S3 contains the Python code.

Supplementary Table S1a. Prespecified MedDRA Preferred Terms, with frequency among the 928 reports, clinical category, and MedDRA primary System Organ Class

Preferred Term	n	% of reports	Category	MedDRA primary SOC
Dizziness	279	30.1	Neurological/vestibular	Nervous system disorders
Anxiety	153	16.5	Anxiety and mood	Psychiatric disorders
Agitation	96	10.3	Behavioral	Psychiatric disorders
Insomnia	91	9.8	Sleep	Psychiatric disorders
Depression	89	9.6	Anxiety and mood	Psychiatric disorders
Amnesia	82	8.8	Cognitive	Nervous system disorders
Disorientation	37	4.0	Cognitive	Psychiatric disorders
Vertigo	35	3.8	Neurological/vestibular	Ear and labyrinth disorders
Discomfort	19	2.0	Nonspecific	General disorders and administration site conditions
Mood swings	16	1.7	Anxiety and mood	Psychiatric disorders
Restlessness	13	1.4	Behavioral	Psychiatric disorders
Nervousness	8	0.9	Anxiety and mood	Psychiatric disorders
Delusion	6	0.6	Psychotic	Psychiatric disorders
Hostility	2	0.2	Behavioral	Psychiatric disorders
Panic disorder	1	0.1	Anxiety and mood	Psychiatric disorders
Impatience	1	0.1	Behavioral	Psychiatric disorders
Social anxiety disorder	0	0.0	Anxiety and mood	Psychiatric disorders

Social anxiety disorder was prespecified but was not recorded among the included reports.

Supplementary Table S1b. Identification and classification of levetiracetam and of recorded antidepressants and antipsychotics

Drug group	Class	Active ingredients
Levetiracetam (index drug)		DRUGNAME field together with the active ingredient field (PROD_AI = levetiracetam); all brand and generic names (e.g., Keppra, Keppra XR, E Keppra, generic levetiracetam); primary suspect drug (ROLE_COD = PS)
Antidepressant	SSRIs	citalopram, escitalopram, fluoxetine, fluvoxamine, paroxetine, sertraline, vortioxetine
Antidepressant	SNRIs	desvenlafaxine, duloxetine, venlafaxine
Antidepressant	TCAs	amitriptyline, clomipramine, doxepin, imipramine, nortriptyline
Antidepressant	Serotonin modulators	trazodone
Antidepressant	Atypical antidepressants	mirtazapine
Antipsychotic	Atypical antipsychotics	aripiprazole, asenapine, cariprazine, clozapine, olanzapine, paliperidone, quetiapine, risperidone
Antipsychotic	Typical antipsychotics	haloperidol, levomepromazine, prochlorperazine (frequently used as an antiemetic)
Removed after manual review		Non-psychotropic drugs captured by name matching: pantoprazole, lansoprazole, propranolol, metoclopramide (flagged as antidepressants; 7 reports) and temozolomide (flagged as antipsychotic; 3 reports)

Brand names recorded in DRUGNAME were mapped to active ingredients (e.g., Zoloft → sertraline, Lexapro and Cipralex → escitalopram, Seroquel → quetiapine, Compazine → prochlorperazine).

Supplementary Table S2a. Complete distribution of reported Preferred Terms within each age group, n (% of the age group)

Preferred Term	Children (n = 35)	Adolescents (n = 56)	Adults (n = 628)	Older adults (n = 209)
Dizziness	3 (8.6%)	14 (25.0%)	183 (29.1%)	79 (37.8%)
Anxiety	1 (2.9%)	6 (10.7%)	116 (18.5%)	30 (14.4%)
Agitation	9 (25.7%)	16 (28.6%)	59 (9.4%)	12 (5.7%)
Insomnia	8 (22.9%)	3 (5.4%)	60 (9.6%)	20 (9.6%)
Depression	2 (5.7%)	9 (16.1%)	60 (9.6%)	18 (8.6%)
Amnesia	2 (5.7%)	2 (3.6%)	57 (9.1%)	21 (10.0%)
Disorientation	1 (2.9%)	1 (1.8%)	25 (4.0%)	10 (4.8%)
Vertigo	2 (5.7%)	1 (1.8%)	27 (4.3%)	5 (2.4%)
Discomfort	0 (0.0%)	3 (5.4%)	15 (2.4%)	1 (0.5%)
Mood swings	5 (14.3%)	0 (0.0%)	11 (1.8%)	0 (0.0%)
Restlessness	2 (5.7%)	1 (1.8%)	3 (0.5%)	7 (3.3%)
Nervousness	0 (0.0%)	0 (0.0%)	3 (0.5%)	5 (2.4%)
Delusion	0 (0.0%)	0 (0.0%)	5 (0.8%)	1 (0.5%)
Hostility	0 (0.0%)	0 (0.0%)	2 (0.3%)	0 (0.0%)
Impatience	0 (0.0%)	0 (0.0%)	1 (0.2%)	0 (0.0%)
Panic disorder	0 (0.0%)	0 (0.0%)	1 (0.2%)	0 (0.0%)

Monte Carlo chi-square test for the overall association between age group and Preferred Term (10,000 permutations): $p < 0.001$.

Supplementary Table S2b. Adjusted odds ratios for each Preferred Term by age group (reference: adults), adjusted for sex and psychotropic co-medication

Preferred Term	Reports	Children aOR (95% CI)	p	q	Adolescents aOR (95% CI)	p	q	Older adults aOR (95% CI)	p	q
Dizziness	279	0.21 (0.06–0.71)	0.012	0.119	0.74 (0.39–1.39)	0.346	0.603	1.48 (1.06–2.07)	0.020	0.119
Anxiety	153	0.15 (0.02–1.11)	0.063	0.190	0.62 (0.26–1.50)	0.294	0.587	0.76 (0.49–1.17)	0.213	0.512
Agitation	96	2.56 (1.13–5.83)	0.025	0.119	3.22 (1.67–6.20)	<0.001	0.011	0.54 (0.28–1.03)	0.062	0.190
Insomnia	91	2.87 (1.22–6.74)	0.015	0.119	0.57 (0.17–1.89)	0.355	0.603	1.00 (0.58–1.71)	0.996	0.996
Depression	89	0.76 (0.18–3.31)	0.718	0.821	2.29 (1.05–5.02)	0.038	0.153	0.95 (0.55–1.66)	0.864	0.901
Amnesia	82	0.58 (0.14–2.52)	0.472	0.689	0.36 (0.09–1.53)	0.166	0.443	1.11 (0.65–1.88)	0.703	0.821
Disorientation	37	0.76 (0.10–5.87)	0.789	0.861	0.49 (0.06–3.72)	0.488	0.689	1.22 (0.57–2.59)	0.608	0.769
Vertigo	35	1.52 (0.34–6.89)	0.585	0.769	0.40 (0.05–3.04)	0.377	0.603	0.56 (0.21–1.49)	0.249	0.542
Discomfort	19	not estimable			not estimable			not estimable		

Preferred Term	Reports	Children aOR (95% CI)	p	q	Adolescents aOR (95% CI)	p	q	Older adults aOR (95% CI)	p	q
Mood swings	16	not estimable			not estimable			not estimable		
Restlessness	13	not estimable			not estimable			not estimable		
Nervousness	8	not estimable			not estimable			not estimable		
Delusion	6	not estimable			not estimable			not estimable		
Hostility	2	not estimable			not estimable			not estimable		
Impatience	1	not estimable			not estimable			not estimable		
Panic disorder	1	not estimable			not estimable			not estimable		

aOR, adjusted odds ratio; q, Benjamini–Hochberg false discovery rate–adjusted p-value across the 24 estimable contrasts.

Models were fitted for Preferred Terms with ≥20 reports.

Supplementary Table S3. Reported Preferred Terms by sex, n (% of sex group)

Preferred Term	Female (n = 565)	Male (n = 363)	Test	p	q
Dizziness	187 (33.1%)	92 (25.3%)	Chi-square	0.012	0.064
Anxiety	91 (16.1%)	62 (17.1%)	Chi-square	0.696	0.884
Agitation	42 (7.4%)	54 (14.9%)	Chi-square	<0.001	0.004
Insomnia	49 (8.7%)	42 (11.6%)	Chi-square	0.147	0.358
Depression	62 (11.0%)	27 (7.4%)	Chi-square	0.074	0.297
Amnesia	49 (8.7%)	33 (9.1%)	Chi-square	0.827	0.945
Disorientation	20 (3.5%)	17 (4.7%)	Chi-square	0.385	0.616
Vertigo	29 (5.1%)	6 (1.7%)	Chi-square	0.007	0.053
Discomfort	14 (2.5%)	5 (1.4%)	Chi-square	0.248	0.441
Mood swings	7 (1.2%)	9 (2.5%)	Chi-square	0.157	0.358
Restlessness	5 (0.9%)	8 (2.2%)	Chi-square	0.095	0.305
Nervousness	4 (0.7%)	4 (1.1%)	Fisher	0.718	0.884
Delusion	2 (0.4%)	4 (1.1%)	Fisher	0.217	0.434
Hostility	2 (0.4%)	0 (0.0%)	Fisher	0.523	0.761
Impatience	1 (0.2%)	0 (0.0%)	Fisher	1.000	1.000
Panic disorder	1 (0.2%)	0 (0.0%)	Fisher	1.000	1.000

q, Benjamini–Hochberg adjusted across the 16 comparisons.

Supplementary Table S4a. Reports with (n = 230) and without (n = 698) a recorded antidepressant or antipsychotic: crude odds ratios and odds ratios adjusted for age and sex

Preferred Term	Antidepressant and/or antipsychotic, n (%)	Neither class, n (%)	Crude OR	p (Fisher exact)	aOR (95% CI)	p (adjusted model)	q
Dizziness	51 (22.2%)	228 (32.7%)	0.59	0.003	0.52 (0.37–0.75)	<0.001	0.001
Anxiety	57 (24.8%)	96 (13.8%)	2.07	<0.001	2.12 (1.45–3.08)	<0.001	0.001
Agitation	13 (5.7%)	83 (11.9%)	0.44	0.006	0.61 (0.33–1.14)	0.123	0.239
Insomnia	27 (11.7%)	64 (9.2%)	1.32	0.252	1.36 (0.84–2.20)	0.214	0.244
Depression	32 (13.9%)	57 (8.2%)	1.82	0.014	1.78 (1.12–2.85)	0.016	0.042
Amnesia	20 (8.7%)	62 (8.9%)	0.98	1.000	0.96 (0.56–1.63)	0.880	0.880
Disorientation	13 (5.7%)	24 (3.4%)	1.68	0.172	1.67 (0.83–3.37)	0.149	0.239
Vertigo	6 (2.6%)	29 (4.2%)	0.62	0.326	0.55 (0.22–1.34)	0.188	0.244
Discomfort	5 (2.2%)	14 (2.0%)	1.09	0.794			
Mood swings	2 (0.9%)	14 (2.0%)	0.43	0.382			
Restlessness	0 (0.0%)	13 (1.9%)	0.11	0.046			
Nervousness	1 (0.4%)	7 (1.0%)	0.43	0.687			
Delusion	1 (0.4%)	5 (0.7%)	0.61	1.000			
Hostility	0 (0.0%)	2 (0.3%)	0.60	1.000			
Impatience	1 (0.4%)	0 (0.0%)	9.13	0.248			
Panic disorder	1 (0.4%)	0 (0.0%)	9.13	0.248			

Reference group: reports without either class. Crude ORs and Fisher exact p-values correspond to Table 8 of the manuscript, where 95% confidence intervals for the six most frequent terms are reported in the text; crude ORs for terms with a zero cell use a 0.5 continuity correction. aOR, odds ratio adjusted for age (continuous) and sex, fitted for Preferred Terms with ≥20 reports and ≥5 exposed reports; q, Benjamini–Hochberg adjusted across the 8 adjusted models.

Supplementary Table S4b. Sensitivity analysis: the six reports whose only recorded psychotropic was prochlorperazine reclassified as unexposed (224 vs. 704)

Preferred Term	Antidepressant and/or antipsychotic, n (%)	Neither class, n (%)	Crude OR	p (Fisher exact)	aOR (95% CI)	p (adjusted model)	q
Dizziness	51 (22.8%)	228 (32.4%)	0.62	0.006	0.55 (0.38–0.78)	<0.001	0.004
Anxiety	56 (25.0%)	97 (13.8%)	2.09	<0.001	2.14 (1.47–3.12)	<0.001	0.001
Agitation	13 (5.8%)	83 (11.8%)	0.46	0.011	0.63 (0.34–1.18)	0.153	0.307
Insomnia	24 (10.7%)	67 (9.5%)	1.14	0.607	1.17 (0.71–1.93)	0.527	0.602
Depression	32 (14.3%)	57 (8.1%)	1.89	0.009	1.85 (1.16–2.96)	0.010	0.027
Amnesia	20 (8.9%)	62 (8.8%)	1.02	1.000	1.00 (0.59–1.70)	0.997	0.997
Disorientation	11 (4.9%)	26 (3.7%)	1.35	0.434	1.34 (0.65–2.77)	0.434	0.579
Vertigo	6 (2.7%)	29 (4.1%)	0.64	0.422	0.56 (0.23–1.39)	0.212	0.340
Discomfort	5 (2.2%)	14 (2.0%)	1.13	0.790			
Mood swings	2 (0.9%)	14 (2.0%)	0.44	0.383			
Restlessness	0 (0.0%)	13 (1.8%)	0.11	0.046			
Nervousness	1 (0.4%)	7 (1.0%)	0.45	0.688			
Delusion	1 (0.4%)	5 (0.7%)	0.63	1.000			
Hostility	0 (0.0%)	2 (0.3%)	0.63	1.000			
Impatience	1 (0.4%)	0 (0.0%)	9.46	0.241			
Panic disorder	1 (0.4%)	0 (0.0%)	9.46	0.241			

Supplementary Table S4c. Sensitivity analysis: adjusted for age, sex, and body weight

Preferred Term	Antidepressant and/or antipsychotic, n (%)	Neither class, n (%)	Crude OR	p (Fisher exact)	aOR (95% CI)	p (adjusted model)	q
Dizziness	51 (22.2%)	228 (32.7%)	0.59	0.003	0.50 (0.35–0.72)	<0.001	0.001
Anxiety	57 (24.8%)	96 (13.8%)	2.07	<0.001	2.01 (1.38–2.94)	<0.001	0.001
Agitation	13 (5.7%)	83 (11.9%)	0.44	0.006	0.68 (0.36–1.28)	0.233	0.280
Insomnia	27 (11.7%)	64 (9.2%)	1.32	0.252	1.43 (0.87–2.33)	0.155	0.248
Depression	32 (13.9%)	57 (8.2%)	1.82	0.014	1.75 (1.09–2.81)	0.020	0.054
Amnesia	20 (8.7%)	62 (8.9%)	0.98	1.000	0.94 (0.55–1.60)	0.814	0.814
Disorientation	13 (5.7%)	24 (3.4%)	1.68	0.172	1.68 (0.83–3.41)	0.148	0.248
Vertigo	6 (2.6%)	29 (4.2%)	0.62	0.326	0.58 (0.24–1.45)	0.245	0.280
Discomfort	5 (2.2%)	14 (2.0%)	1.09	0.794			
Mood swings	2 (0.9%)	14 (2.0%)	0.43	0.382			
Restlessness	0 (0.0%)	13 (1.9%)	0.11	0.046			
Nervousness	1 (0.4%)	7 (1.0%)	0.43	0.687			
Delusion	1 (0.4%)	5 (0.7%)	0.61	1.000			
Hostility	0 (0.0%)	2 (0.3%)	0.60	1.000			
Impatience	1 (0.4%)	0 (0.0%)	9.13	0.248			
Panic disorder	1 (0.4%)	0 (0.0%)	9.13	0.248			

Supplementary Table S4d. Sensitivity analysis: adjusted for age group and sex

Preferred Term	Antidepressant and/or antipsychotic, n (%)	Neither class, n (%)	Crude OR	p (Fisher exact)	aOR (95% CI)	p (adjusted model)	q
Dizziness	51 (22.2%)	228 (32.7%)	0.59	0.003	0.53 (0.37–0.75)	<0.001	0.002
Anxiety	57 (24.8%)	96 (13.8%)	2.07	<0.001	1.95 (1.34–2.84)	<0.001	0.002
Agitation	13 (5.7%)	83 (11.9%)	0.44	0.006	0.57 (0.31–1.06)	0.077	0.154
Insomnia	27 (11.7%)	64 (9.2%)	1.32	0.252	1.42 (0.87–2.32)	0.159	0.202
Depression	32 (13.9%)	57 (8.2%)	1.82	0.014	1.87 (1.16–3.01)	0.010	0.028
Amnesia	20 (8.7%)	62 (8.9%)	0.98	1.000	0.92 (0.54–1.57)	0.768	0.768
Disorientation	13 (5.7%)	24 (3.4%)	1.68	0.172	1.68 (0.83–3.40)	0.151	0.202
Vertigo	6 (2.6%)	29 (4.2%)	0.62	0.326	0.54 (0.22–1.32)	0.176	0.202
Discomfort	5 (2.2%)	14 (2.0%)	1.09	0.794			
Mood swings	2 (0.9%)	14 (2.0%)	0.43	0.382			
Restlessness	0 (0.0%)	13 (1.9%)	0.11	0.046			
Nervousness	1 (0.4%)	7 (1.0%)	0.43	0.687			
Delusion	1 (0.4%)	5 (0.7%)	0.61	1.000			
Hostility	0 (0.0%)	2 (0.3%)	0.60	1.000			
Impatience	1 (0.4%)	0 (0.0%)	9.13	0.248			
Panic disorder	1 (0.4%)	0 (0.0%)	9.13	0.248			
